# 3D Histopathology—a Lung Tissue Segmentation Workflow for Microfocus X-ray-Computed Tomography Scans

**DOI:** 10.1007/s10278-017-9966-5

**Published:** 2017-03-24

**Authors:** Lasse Wollatz, Steven J. Johnston, Peter M. Lackie, Simon J. Cox

**Affiliations:** 1 Faculty of Engineering and the Environment, University of Southampton, Southampton, SO17 1BJ UK; 2 Faculty of Medicine, University of Southampton, Southampton, SO17 1BJ UK

**Keywords:** Lung, Image segmentation, Computed tomography, Histopathology, ImageJ, Vascular network

## Abstract

Lung histopathology is currently based on the analysis of 2D sections of tissue samples. The use of microfocus X-ray-computed tomography imaging of unstained soft tissue can provide high-resolution 3D image datasets in the range of 2–10 μm without affecting the current diagnostic workflow. Important details of structural features such as the tubular networks of airways and blood vessels are contained in these datasets but are difficult and time-consuming to identify by manual image segmentation. Providing 3D structures permits a better understanding of tissue functions and structural interrelationships. It also provides a more complete picture of heterogeneous samples. In addition, 3D analysis of tissue structure provides the potential for an entirely new level of quantitative measurements of this structure that have previously been based only on extrapolation from 2D sections. In this paper, a workflow for segmenting such 3D images semi-automatically has been created using and extending the ImageJ open-source software and key steps of the workflow have been integrated into a new ImageJ plug-in called LungJ. Results indicate an improved workflow with a modular organization of steps facilitating the optimization for different sample and scan properties with expert input as required. This allows for incremental and independent optimization of algorithms leading to faster segmentation. Representation of the tubular networks in samples of human lung, building on those segmentations, has been demonstrated using this approach.

## Introduction

**Fig. 1:**
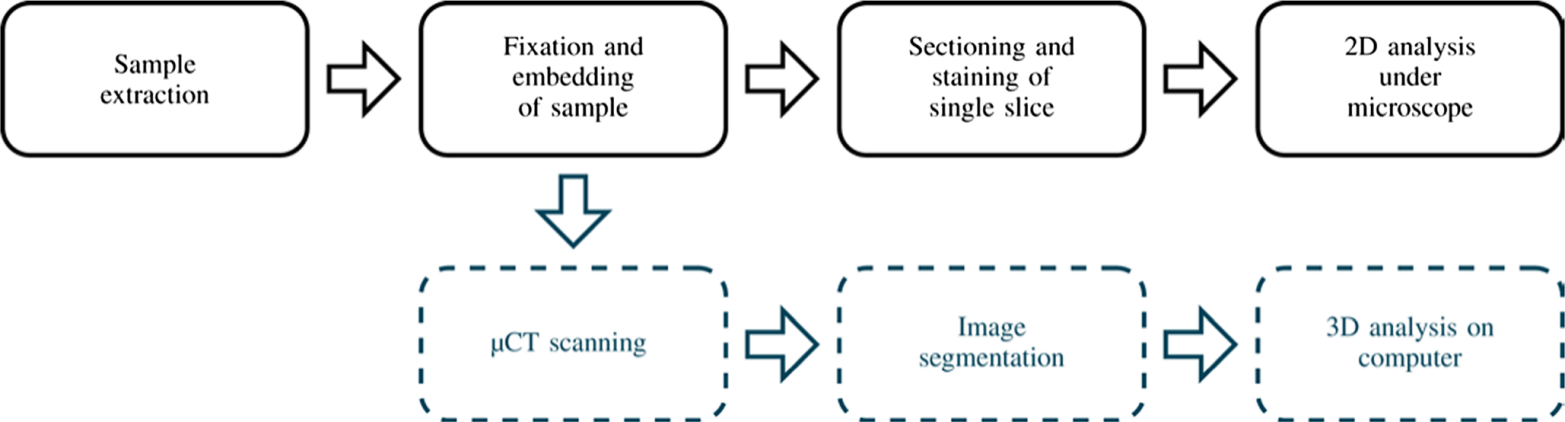
Current histology workflow (*top*) and proposed workflow (*bottom*). As μCT is non-invasive, this can be seen as an additional approach

Histopathology provides structural details of tissue samples on a cellular level allowing disease-associated changes to be identified. It offers a key diagnostic tool for fibrotic lung diseases, particularly those that cannot be clearly identified on the basis of patient-computed tomography (CT) or high-resolution CT [1–4] and is often regarded as the gold standard [5, 6]. For routine histopathology, surgical biopsies are taken from a patient, providing three-dimensional (3D) tissue samples that are chemically fixed to preserve tissue structure and then embedded in wax to allow for histological sectioning. For diagnostic purposes, sections are stained to identify the overall tissue structure and highlight certain tissue components before analysis under a microscope by a trained histopathologist (see Fig. 1). These microscopy images are increasingly provided through digital scanning of tissue slices. This allows analysis based on the digital image (virtual microscopy). Systems for computer-assisted diagnosis (CAD) used to highlight relevant features or suggest a possible diagnosis are also becoming available [5, 7].

The established methods in histopathology have proven to be critical to the diagnosis of many lung diseases. There are some diseases like idiopathic pulmonary fibrosis, non-specific interstitial pneumonia, or extrinsic allergic alveolitis, where diagnosis is difficult and an agreement between histopathologists is generally only fair to moderate (kappa agreement coefficients ranging from 0.2 to 0.7) [8]. By producing a three-dimensional scan of the sample, additional information about the tissue will be gained [9]. Instead of single slices, 100 to 1000 times the number of slices can be viewed, at various slice orientations. This aids the identification of rarer structural changes and reveals the degree of heterogeneity in tissue structure. Further benefits include analysis of 3D structures, specifically 3D networks, revealing tissue function as well as interrelationships between objects [9, 10], enabling the application of established stereological methods [11].

Microfocus X-ray-computed tomography (μCT) of soft tissue embedded in wax produces relatively high resolution (~7 μm or better), but low-contrast 3D images. Identification of key features inside these images is challenging but important. Image segmentation describes this process of distinguishing between the areas of interest in an image and the remaining area. Parts of the image, which are not of interest, are commonly removed or areas of different features are marked with different (digital) labels. Segmenting some features such as the walls of the airways and blood vessels manually is effectively impossible; manual segmentation of the lumen, while possible [12], is time-consuming, taking weeks or months per dataset. Automatic or semi-automatic segmentation is therefore required to make this a useable method for research or diagnosis.

Many methods [13–17] have been developed for automatic segmentation of images in medicine. Standard approaches such as intensity thresholding [18], watershed algorithms [19] and contour region growing [15] do not have the required sensitivity or specificity when used for noisy low-contrast images like soft tissue. Atlas-based approaches [20] require an ideal model which cannot be created for small and very variable structures like the alveoli. Machine learning approaches can reduce the amount of human input and are flexible enough to deal with low-contrast images. They have previously been used for computer-aided diagnosis in histology and for airway detection in lung patient CT [21, 22].

We present here a workflow that allows semi-automated segmentation of airways and blood vessels and its implementation as LungJ using the free, open-source software ImageJ [23]. The workflow is scalable to large 3D μCT scans and targets lung tissue samples. ‘Materials and Methods’ provides details on sample preparation and image acquisition. The procedural steps of the image-processing workflow itself are presented in ‘The Workflow’. Background and choices made for individual workflow procedures are explained for each step. A short summary of the workflow is provided in the ‘Conclusions’.

## Materials and Methods

A number of steps were required before the digital segmentation: Tissue was prepared for scanning, while scanning conditions were optimized to provide the best possible resolution and contrast for the tissue. Scans also had to be pre-processed before segmentation.

### Sample Preparation

Surgically resected human lung tissue was obtained with written informed consent from patients undergoing elective surgery at the University Hospital Southampton, UK. Ethical approval was given from the National Research Ethics Service Committee, South Central-Southampton A, number 08/H0502/32. A representative sample was taken from a macroscopically healthy portion of a lung of a 75-year-old male non-smoker with GOLD stage 0 chronic obstructive pulmonary disease undergoing resection for lung cancer. The samples were fixed with para-formaldehyde in phosphate buffer for 8 h and embedded in wax following a routine histology protocol using a Shandon Hypercentre tissue processor (Fisher Scientific, Loughborough, UK). This resulted in blocks of about 1.5 cm^3^ which were then scanned without any further treatment.

### Image Acquisition and Pre-processing

Samples were scanned using a custom-built Nikon Metrology μCT scanner (μ-VIS, Southampton, UK). An electron-accelerating voltage of 50 kV was used, with a molybdenum reflection target, yielding a mean energy in the order of 18 keV, along with characteristic peaks around 17 and 19 keV. No filtration was used. A beam current of approximately 150 μA was selected, yielding a total beam energy below 8 W. Reconstructions with voxel resolution of 8 μm were created, using standard filtered-back projection (Ram-Lak filter) within the Nikon CT Pro 2.0 package using 3142 projections (360° rotation in 0.11° increments). Typical datasets included 1800 × 2000 × 2000 isotropic voxels. The value of each voxel corresponds to the density of the material at that position and was acquired at 32-bit resolution. As a comparison, hospital CTs acquire 512 × 512 × 80 voxel datasets at 16-bit resolution.

## The Workflow

In order to simplify the workflow and avoid repetitive steps, a software plug-in for ImageJ was developed. This plug-in, called LungJ, had to be capable of handling the large data size of individual scans and segmenting images in a way that is meaningful to and can be interpreted by the final user. An image segmentation method had to be established, and images were post-processed to remove noise and artifacts. Finally, representation of the 3D image data was explored to ensure better understanding of the underlying structure. Each of these distinct steps is detailed below, highlighting important decisions made while creating this workflow.

### Choice of Software

It was important that the software package of choice, which a plug-in would be programmed for, had to be one that was familiar to potential users. This would maximize usability, especially for users less familiar with image processing. Several software packages were evaluated for implementation of this method [24]. Paid software, such as Amira 3D [25] or OsiriX [26], was not user configurable to the required degree or limited to certain operating platforms. Table 1 shows that ImageJ [23, 27] is, together with Matlab, the most widely used image-processing software in medical research. ImageJ runs in Java and is therefore platform independent. It has the advantage over Matlab of being open-source freeware. The pre-packaged Fiji (Fiji Is Just ImageJ) distribution of ImageJ also comes with a plug-in for machine learning-based image segmentation.

**Table 1:** Popularity of different imaging software in research, based on results from 15th May 2016 (www.ncbi.nlm.nih.gov/gquery and scholar.google.com). Search results for Amira 3D exclude authors with the name Amira

Software	Type	Developer	PubMed central papers	Google scholar results
ImageJ	Open Source	Wayne Rasband	75,400	157,000
MATLAB	Commercial	Mathworks	67,305	2,010,000
NIS-Elements	Commercial	Nikon	6184	18,000
Imaris	Commercial	Bitplane	4842	12,300
Amira 3D	Commercial	FEI	1903	13,800
SlideBook	Commercial	3i	2807	6010
ImagePro Plus	Commercial	MediaCybernetics	2464	5420
OsiriX	Commercial (free version available)	Pixmeo	1515	9320
CellProfiler	Open source	CellProfiler Team, MIT	1055	3350
ITK-SNAP	Freeware	University of Pennsylvania and University of Utah	505	2610
Avizo 3D	Commercial	FEI	371	4210
VGStudio	Commercial	Volume graphics	125	1620
Vaa3D	Open source	Hanchuan Peng and Howard Hughes	77	216
BioImageXD	Open source	Pasi Kankaanpää, Lassi Paavolainen, Varpu Marjomäki, Jyrki Heino et al.	69	241

Based on the framework provided by Fiji, a plug-in was developed to tackle the different tasks of image segmentation. LungJ [28] tools include a function for segmenting an image, as well as tools for pre- and post-processing the image. While all these processes can be done in Fiji, the tools provide important simplifications, as they reduce the amount of knowledge a user needs to have about the image (e.g. bit-depth or absolute values of voxels). Instead of opening the image, launching the Trainable WEKA Segmentation (TWS) [29], loading the classifier, applying it and then filtering out the relevant mask, with LungJ all of this can be done from a single graphical user interface (GUI) which in turn calls the relevant TWS functions. The overall structure of LungJ is very modular and therefore adaptive, in order to align with the ImageJ philosophy. While the original scan can be in any format handled by ImageJ, LungJ saves intermediate steps as loss-less tagged image files. By combining multiple modular functions in one GUI and allowing default settings to be defined, it was possible to reduce the number of steps of the workflow.

### Handling Large Images

The output image volumes were several tens of gigabytes each. Image processing requires multiple times the memory size of an image, i.e. several tens to hundreds of GB random access memory (RAM). In order to be able to process these large image files, they were split into smaller sub-volumes and processed separately, enabling us to complete processing on a computer with only 16GB of RAM while still allowing 3D interrelationships with the volume to be addressed. Currently available methods, like the virtual image stack, do not allow custom-sized image blocks and tests; using them, in conjunction with the image segmentation algorithm shown in the next section, caused an out-of-memory error. The image blocks are loaded individually and only on demand. Global properties of the image were saved in a separate file and used during further processing steps to ensure consistency of intensity values over the whole volume. This introduced a novel image representation method which required ImageJ to only hold a directory path instead of loading a full image. Using this representation, any available ImageJ filter can be applied to the whole image. Furthermore, image properties such as the intensity distribution histogram can be produced. The use of smaller image blocks also enables future adaption of this process in parallel computing or cloud computing.

**Fig. 2:**
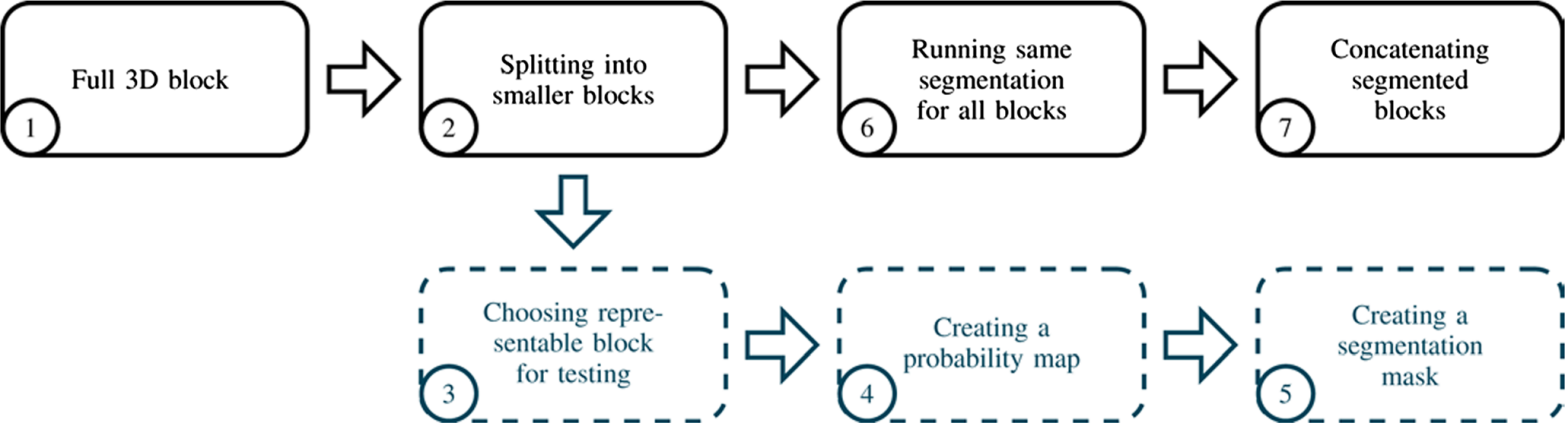
Modular approach of LungJ solving several problems of the image segmentation

Three functions for creating, processing and concatenating image sub-volumes were implemented. They form the backbone of the sub-volume image processing. As shown in Fig. 2, an image was split prior to the segmentation and a representable block was chosen to test the segmentation. This enabled faster and hence more efficient testing and troubleshooting of a segmentation technique compared to running a segmentation on a full scan. Once the segmentation procedure had been established, it was applied to all the sub-volumes by processing each one in turn. Finally, the sub-volumes were assembled into a single image by applying the concatenating function.

A further addition was the use of halos enclosing the image blocks, as commonly used in parallel computing. The use of halos avoided boundary artifacts, and a function for halo exchange was implemented. Cubical sub-volumes were used when applying three-dimensional processing methods because of their smaller surface area compared to other cuboids.

**Fig. 3:**
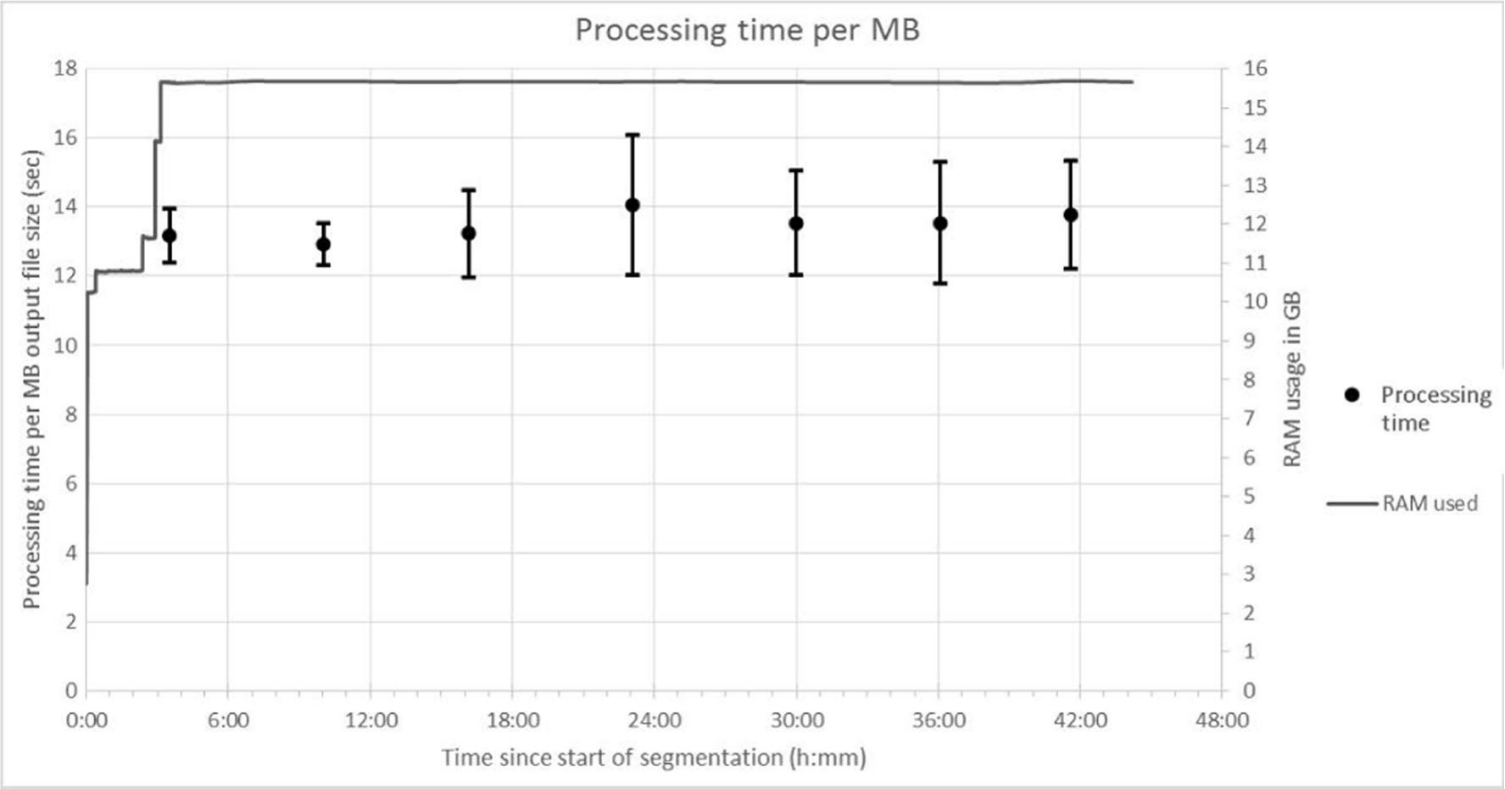
Processing speed of blocks based on the slowest of the runs (44 h, Intel Pentium processor). The average processing speed over 35 consecutive blocks was taken and standard errors calculated. RAM usage is shown for reference

With these tools in place, a segmentation was achieved on an Intel-i7-4770, with 3.40 GHz and 16GB RAM within 12 h whereas manual segmentation of similar images could take between 3 and 6 months. As shown in Fig. 3, for a run on a computer with a slower Intel Pentium G2020T at 2.50 GHz, the processing speed does not change significantly over time despite a memory leak in ImageJ causing full RAM usage.

### Image Segmentation

**Fig. 4:**
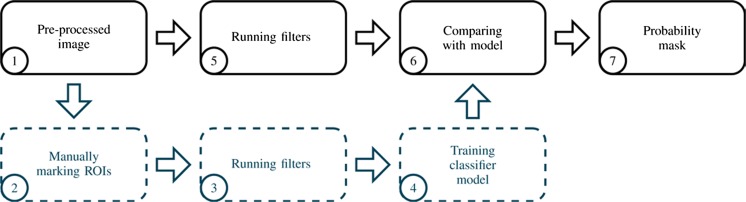
Machine learning algorithm. Based on partial manual segmentation, a classifier model is trained and then applied to the whole image

A machine learning algorithm was applied for the image segmentation. Fiji comes with a segmentation plug-in called Trainable WEKA Segmentation [29]. For the segmentation, the random-forest algorithm was chosen. This algorithm has been used for image segmentation of neuronal processes in biomedical applications as well as segmentation of larger vessels in CT scans of whole lungs before [30, 31]. The application of the machine learning algorithm involved the creation of a classifier model which had to be trained by using reference data and filters, as outlined in Fig. 4. The algorithm could then determine the likelihood of each voxel of further images (or further parts of the same image) belonging to the feature of interest.

As values of the voxels are proportional to the sample’s X-ray absorption, any pre-processing methods affecting the histogram will also lead to a loss of information within the image or relative to other scans. Therefore, no normalization or equalization was performed. This underlines the importance of a standardized sample preparation and scanning procedure to produce comparable images. No noise reduction filter was applied apart from a Gaussian blur prior to other feature detecting filters (outlined in the end of this section).

**Fig. 5:**
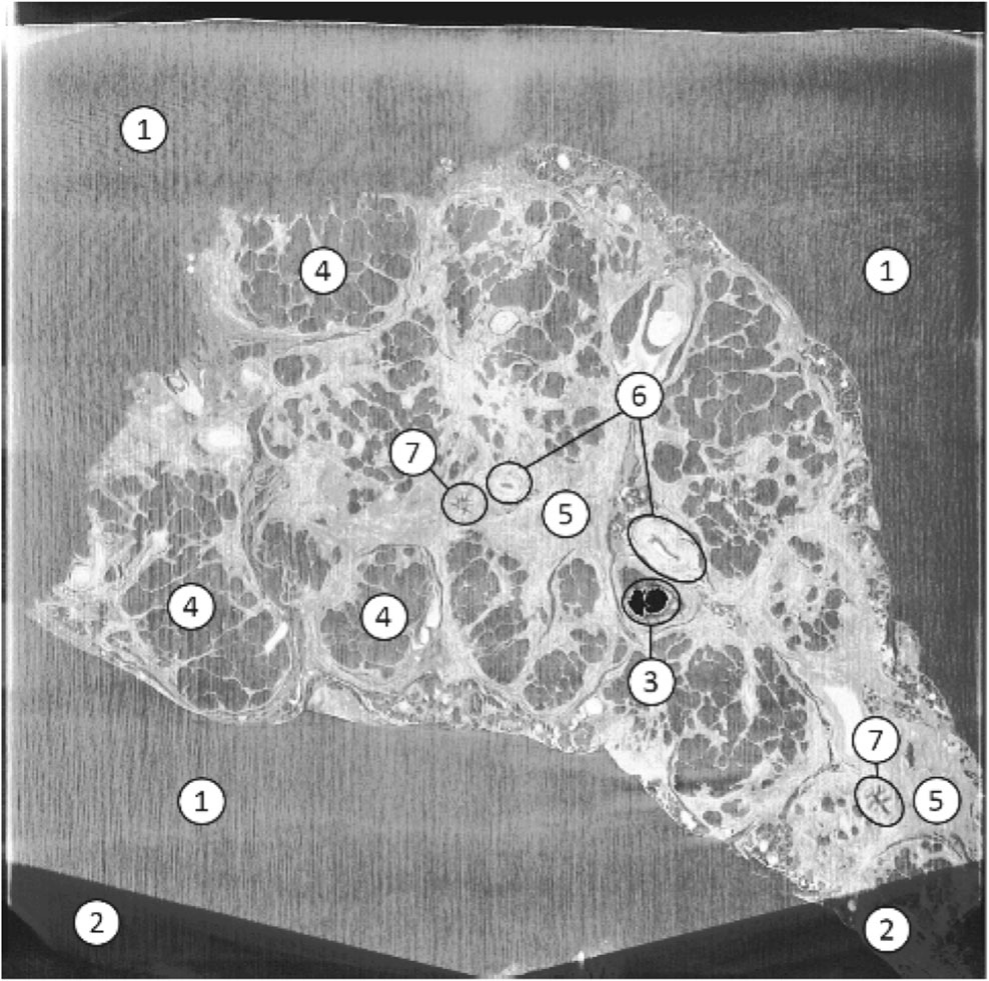
A single section of a μCT scan of wax-embedded human lung tissue showing areas of wax, area outside the scan and distinct features of interest. Features 5, 6 and 7 were targeted for this project. (*1*) Wax/ background. (*2*) Area outside scan. (*3*) Air bubble in wax. (*4*) Alveolar area. (*5*) Connective tissue. (*6*) Blood vessel. (*7*) Airway

Reference data was provided in the form of manually segmented images. For large parts of the image, it was not possible to clearly differentiate and manually segment vessel wall and surrounding tissue. Accordingly, manual training slices were selected, which showed the boundaries of the vessel wall as clearly as possible. Two airways and two blood vessels were selected as foreground, and four representative areas that were not of interest were selected as background. Whilst selection of more training data can improve the classifier model, too much training data in this case resulted in over-segmentation due to the difficulty in accurately selecting training data manually. A selection of a wall including the inside area gave better results, as it included edges which are easier to identify than other features when using filters. The inside of a vessel matched the background, as both were filled with wax and therefore had the same density (see Fig. 5). This caused an over-segmentation of the background which was dealt with during the post-processing detailed in the following section.

**Fig. 6:**
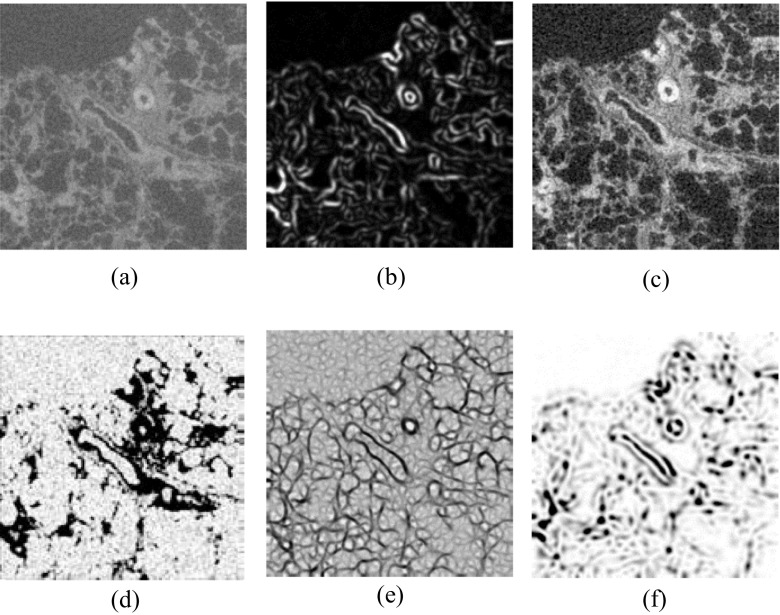
Original image and result of different filters applied. **a** Original image. **b** Structure (eigenvalue). **c** Neighbors. **d** Entropy. **e** Hessian (eigenvalue). **f** Hessian (orientation)

A set of image filters is provided by the TWS, and a selection of these was chosen based on the features to detect. Features of interest are shown in Fig. 5. As key structural elements of the lung, this project focused on detecting blood vessels and airways. This method can also be used to detect other features such as fibrotic structures, as shown in the ‘Data Presentation’ section. Vascular structures are characterized by a bright (X-ray dense) surrounding and a dark (X-ray lucent) centre. These attributes suggested the use of filters targeting edges. The choice of filters was based on literature review as well as screenings of all available filters with a representable image, as shown in Fig. 6. Filters like ‘Structure’, ‘Neighbors’, ‘Entropy’ and ‘Hessian’ were able to separate areas of interest from the rest of the image. The Hessian filter highlights edges and was proposed for tube-like structure detection by Sato et al. [32]. The use of ‘Entropy’ filters for CT images is encouraged by Bae et al. [33].

Next, a classifier model was created. The reference segmentation and the choice of filters were provided to the TWS which trained a classifier model.

Using the TWS fast-random-forest algorithm and ‘Structure’, ‘Neighbors’, ‘Entropy’ and ‘Hessian’ filters, it was possible to train a classifier model from a manual segmentation. This model was used to segment the whole μCT scan. The result was a probability map where higher values represent a larger likelihood of a voxel being an airway or a blood vessel.

### Image Post-processing

The probability map was converted into a binary image (mask), where each voxel was given a value of 0 or 1, representing areas of no interest and areas of interest, respectively. This was done by manually defining and applying a threshold. This mask contained the vessel walls as well as some areas surrounding both the inside and outside of the wall. To remove the voxels from the mask which were not part of the vessel walls, the mask was applied to the original image and then another threshold was applied to remove the darker areas representing wax. This threshold was again chosen manually.

This second mask contained some undesired regions, such as air bubbles or noise. Whilst erode and dilate operations are useful tools to remove noise [34], they do affect the surface of correctly identified regions. Erosion of a mask removes all voxels within a radius from the masks’ surface, while dilation adds voxels within a certain radius from the masks’ surface to the mask. For both operators, the radius is usually equal to the size of one voxel. The combination of one erode operation and one dilate operation of equal radius is referred to as opening, and the opposite action of dilating before eroding is referred to as closing.

**Fig. 7:**
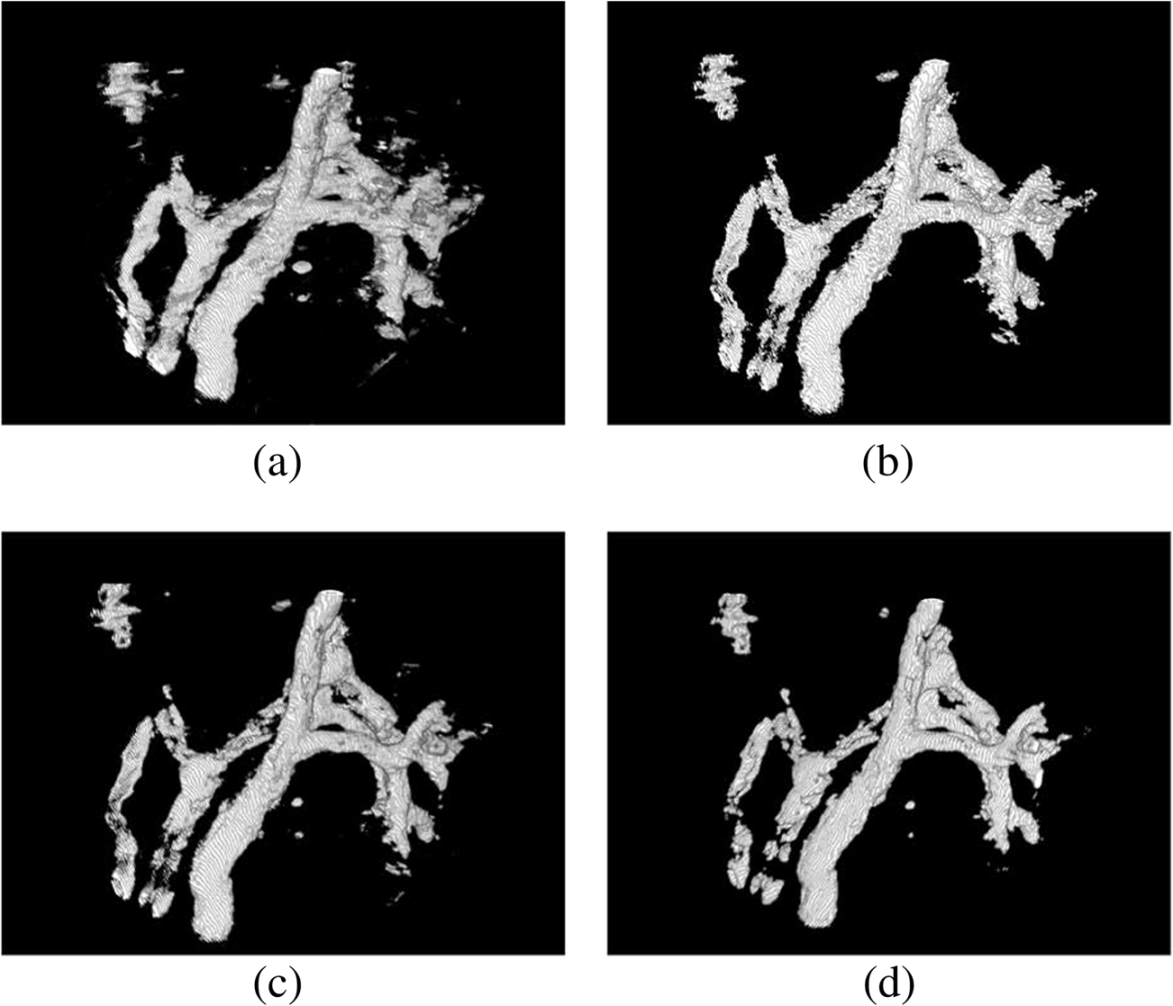
Difference between connected regions and erode/dilate algorithm for noise removal. Note how erosion and dilation either do not remove a lot of noise (**c**) or visibly remove part of the structure of interest (**d**). **a** Original mask. **b** Connected region algorithm with minimum region size 700. **c** Opening (erode, dilate) followed by closing (dilate, erode) operation. **d** Closing, opening and radius 2 opening operation

Erode and dilate algorithms have been used in medical image processing previously [35]. The effect of applying them in comparison to an algorithm identifying and removing small connected regions has been studied. Results of the minimum region size of the connected region algorithm, as well as two different implementations of erode and dilate functions, are presented in Fig. 7b and Fig. 7c, d, respectively. Figure 7c used a modified version of the algorithm presented by Antonelli et al. [34], which has been slightly optimized using the idempotent nature of the operators, whilst the others were based on a set of trial runs.

It was found that algorithms for removing small connected regions reduce the loss of shape but are multiple times more time-consuming (see Table 2), adding about 1 h to the total processing time. Noise removal through removal of ‘small connected regions’ shown in Fig. 7b gives better results than opening and closing operations shown in Fig. 7c, d. The more sophisticated algorithm leaves the actual boundary/surface of the vessels untouched, while erode and dilate operations affect both noise and valid segmentation. For the large connected vessels, erode and dilate are not strong enough for efficient noise removal.

**Table 2:** Timings for two different images on two different machines. Image 1 was 1600 × 1600 × 1200px in size at 16bit and Image 2 1374 × 1497 × 500px at 8bit. Machine 1 had 16GB RAM, 64-bit, Intel Pentium CPU G2020T @2.50 GHz and a local HDD Drive. Machine 2 has 16GB RAM, 64bit Intel i7–4770 @3.40 GHz and a network drive. Two runs were done and averaged for image 2 and one for image 1

Image	Computer	Blocks	Time (h:mm)					
			Block creation	Segmentation	Thresholding	Erode dilate	Connected regions	Total
1	1	245 (no halo)	0:01	44:11	0:04	0:01	0:52	44:17 to 45:08
2	1	72 (16px halo)	0:00 (9 s)	17:59	0:01	0:01	1:14	18:01 to 19:14
1	2	245 (no halo)	0:19	11:38	0:16	0:17	0:42	12:30 to 12:55
2	2	72 (16px halo)	0:04	5:05	0:06	0:06	0:47	5:21 to 6:02

After post-processing of the map, only the vessel walls were left and noise had largely been removed.

### Data Presentation

Once a segmentation mask had been created, a selection of presentation views was generated. This included 2D and projected 3D and 3D data representation [17, 36].

**Fig. 8:**
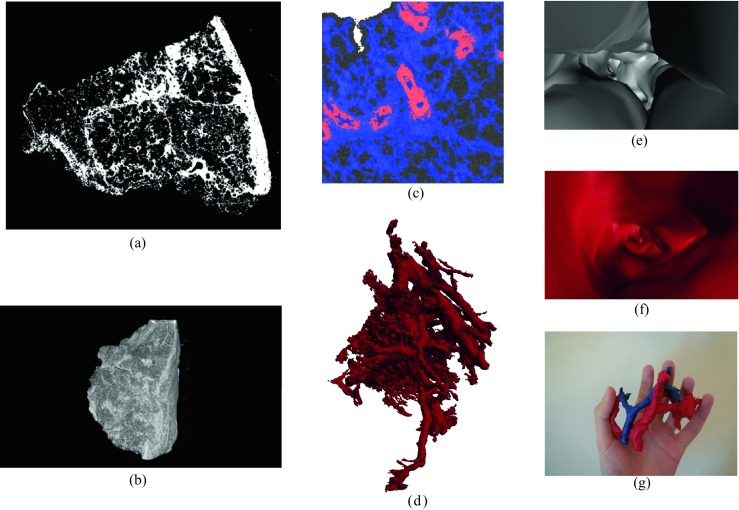
Image representations created after segmenting the whole tissue volume. **a** Fibrotic area of cross-section. **b** 3D view of the fibrotic structure. **c** Colorized overlay of masks on top of original image. **d** 3D view of vessels. **e** 3D render of the inside of an airway. **f** 3D render of the inside of a blood vessel. **g** 3D print in ABS post-painted (airways: *blue*, blood vessels: *red*)

The major advantage of the proposed workflow over established microscopy is the extraction of 3D data. Projected 3D views (2D representations of 3D models) of individual features were created. Fiji 3D allowed the display of interactive 3D surface and volumetric images as in Fig. 8b. More elaborate rendering of 3D surface views and videos was possible by using Avizo. This could not only be outside views (Fig. 8d) but also inside views of blood vessels or airways (Fig. 8e, f, respectively) similar to virtual endoscopy [17]. The inside view shown in Fig. 8e allows clear identification of the airway and can show details about vessel size while removing the complexity of the full network.

Apart from projected 3D images, a physical 3D replica was also created. Conversion of the masks to 3D surface objects was possible using Fiji 3D. The use of Blender allowed smoothing of the surface and creation of printable stereolithography files, one of which was printed using a Tiertime UP! Plus 2 printer (Fig. 8g). 3D printed models improve the understanding of structures beyond the ability of 3D rendered graphics which only show a 2D projection [37].

Having a variety of ways of representing the data improved the insight gained into the 3D structure, since a relation to known representations and a new 3D representation were created.

## Conclusions

Current histopathology relies on a limited 2D analysis of tissue samples that are intrinsically 3D and are affected by local challenges and pathological changes that may be highly heterogeneous at several different length scales. The use of 3D scanning techniques to accompany current histology procedures can improve insight into tissue structures and, accordingly, understanding and diagnosis of chronic lung diseases. The workflow for segmentation of multi-gigabyte μCT scans of lung tissue presented in this paper demonstrates this alternative method. It was found that splitting the large μCT images into smaller blocks permits handling of 3D images without requiring unreasonably powerful computers. At the same time, it enables fast testing of a segmentation strategy. Trainable image segmentation using ‘Structure’, ‘Neighbors’, ‘Entropy’ and ‘Hessian’ filters provided good results with vessels being clearly distinguishable. The connected regions algorithm was shown to be the more accurate noise removal algorithm compared to erode and dilate operations. Different ways of data representation were discussed including 2D color maps, 3D rendering and 3D printing. The workflow was implemented in the popular image-processing software ImageJ in a modular fashion which allows for optimization of each single part of the workflow.

Using this method, tubular structures were successfully segmented from an embedded tissue sample without making assumptions about the 3D structure beforehand. Not only was it possible to reduce the processing time from months of manual segmentation to half a day to 2 days of semi-automated segmentation, but also to be able to scale it to volumes of many tens of GBs without reduction in processing speed. The results clearly identified a 3D network of vessels, and various display methods were used to reveal more information about the tissue than previously possible through microscopy.

Another advantage of the proposed method is its implementation as a modular workflow in open-source software. This allows individual segments of the image processing to be improved and optimized as required without revising the whole protocol. User input for training of the algorithm allows flexible interactive selection of features of interest. Reduction of the number of user steps required to operate LungJ allows much easier application of this method in medical research. The workflow has been designed to aid users, provide feedback of success and facilitate visual presentation of results.

As no other workflows for this task are known to the authors, evaluating the method empirically is difficult [38]. Method evaluation can be achieved using unsupervised image segmentation evaluation methods [39]. The modular approach enables the proposed workflow to be adapted to other types of tissue or image conditions. Having this workflow in place shifts focus towards the efficiency of individual modules in the future. The implementation of this workflow in an end-to-end system, including dataset management [40], is also envisioned. Furthermore, we hope to be able to apply this method to images of different diseases to identify parameters of importance and in the long run improve the diagnostic certainty of histopathology.
